# eHealth Literacy Interventions for Older Adults: A Systematic Review of the Literature

**DOI:** 10.2196/jmir.3318

**Published:** 2014-11-10

**Authors:** Ivan Watkins, Bo Xie

**Affiliations:** ^1^School of InformationThe University of Texas at AustinAustin, TXUnited States; ^2^School of Nursing & School of InformationThe University of Texas at AustinAustin, TXUnited States

**Keywords:** health literacy, aging, computers

## Abstract

**Background:**

eHealth resources offer new opportunities for older adults to access health information online, connect with others with shared health interests, and manage their health. However, older adults often lack sufficient eHealth literacy to maximize their benefit from these resources.

**Objective:**

This review evaluates the research design, methods, and findings of eHealth literacy interventions for older adults.

**Methods:**

A systematic review of peer-reviewed research articles from 28 databases in 9 fields was carried out in January 2013. Four rounds of screening of articles in these databases resulted in a final sample of 23 articles.

**Results:**

Findings indicated a significant gap in the literature for eHealth literacy interventions evaluating health outcomes as the outcome of interest, a lack of theory-based interventions, and few studies applied high-quality research design.

**Conclusions:**

Our findings emphasize the need for researchers to develop and assess theory-based interventions applying high-quality research design in eHealth literacy interventions targeting the older population.

## Introduction

### Overview

Electronic health information plays a growing role in how individuals manage their health and interact with the health care system [1]. Online tools enable individuals to connect with others sharing similar health interests [2], participate in interventions [3], or find health services [4]. For instance, 72% of adult Internet users in the United States (US) have searched for health information online, while 35% of *all* US adults diagnosed a health condition online [1]. eHealth resources can help older adults manage chronic health issues, make informed health decisions, or communicate with their providers [3,4].

Problematically, older adults often lack the skills and knowledge necessary to use online health resources [5], and disability, chronic disease, or handicaps can make technology difficult to use. eHealth literacy refers to the “set of skills and knowledge that are essential for productive interactions with technology-based health tools” [6]. While 59% of adults age 65 and above go online, almost 29% of adults 65 and older perceived that a disability or chronic disease made technology use difficult [7], and only 3% of older adults have proficient health literacy [8]. Lower age and higher educational attainment correlate to higher eHealth literacy [9], suggesting that lower socioeconomic status (SES) older adults are particularly susceptible to low eHealth literacy. This disparity is significant because recent evidence indicates low health literacy correlates to poor health outcomes [10].

eHealth literacy interventions offer one solution for increasing older adults’ ability to access and use eHealth resources such as electronic health records, patient portals, online support groups, and self-management tools [3,4]. Prior reviews examined health literacy interventions for older adults [11], eHealth literacy among younger adults [12], health and eHealth literacy combined [13], and online health literacy interventions for all age groups that use experimental designs [14]. However, no known article has systematically reviewed eHealth literacy interventions for older adults. This article addresses this gap in the literature by providing a systematic review of the literature on eHealth literacy interventions for older adults.

### eHealth and Health Literacy

Health literacy is “the degree to which individuals [can] obtain, process, and understand basic health information and services needed to make appropriate health decisions” [15]. This definition of health literacy contains two important elements: an individual’s ability to (1) comprehend health information, and (2) make appropriate decisions with health information. Health literacy evolved from the two distinct perspectives of clinical care and public health [16]. The clinical perspective positions health literacy as a causal factor that influences health outcomes [16]. From this perspective, poor health literacy influences patients’ adherence to clinical recommendations, which affects clinical outcomes [16]. In contrast, the public health perspective situates health literacy as an outcome of interest [16].

Building on the concept of health literacy, eHealth literacy emphasizes information and communication technologies’ (ICTs) growing role in health information. Examples of ICTs relevant to individuals’ health management include patient portals, telehealth systems, and online support systems. eHealth literacy requires a mix of health, information, scientific, media, computer, and Internet literacy [6]. Given ICTs’ rapid development, the skills, knowledge, and literacies that constitute eHealth literacy continually evolve [17]. As a result, individuals must continue to develop their skills and knowledge to maintain their eHealth literacy.

In this new (but growing) field, few studies have yet developed and tested eHealth literacy specific theories. Rather, eHealth interventions often use learning theory to guide interventions (eg, Xie [18-20]). These interventions consistently prove effective at improving older adults’ eHealth literacy, but their results suggest further theoretical development is necessary to advance the field. For instance, Xie [18,20] found no significant difference for learning outcomes between collaborative and individualistic learning conditions, despite the prediction of social interdependence theory (SIT) that suggested the superiority of collaborative learning over individualistic learning. Similarly, a cognitive theory of multimedia learning (CTML) predicts that tutorials presenting information in one modality (eg, visual only) should outperform tutorials presenting redundant information in multiple modalities (eg, visual and audio) [21]. However, an intervention testing this hypothesis with an eHealth tutorial for older adults found no significant difference for learning outcomes between two presentation methods (visual and audio; visual only) [20]. In both examples of interventions guided by learning theory, outcomes did not align with predicted outcomes, suggesting further theoretical development is necessary.

### Aging-Related Issues

Older adults’ distinct characteristics may explain why learning theories have not generalized to eHealth literacy interventions. Cognitive aging examines age-related changes in cognition, such as reduced information processing speed or a diminished ability to coordinate and integrate information [22]. Cognitive aging studies consistently find negative linear associations between chronological age and cognitive performance [22]. Learning theories developed with younger adults (eg, SIT and CTML) do not account for the influence of cognitive aging, which may explain why these theories have not generalized to older adults. For instance, a Web-based tutorial that provides redundant information (eg, visual text and audio narration that present identical instructional content) may help compensate for age-related declines in working memory [23,24]. However, CTML does not account for the effects of cognitive aging and predicts redundant information decreases learning outcomes [25].

Diversity within the older population may also affect intervention outcomes. This diversity includes chronological age, along with race and ethnicity. Chronological age can range from 50 to over 100, while racial and ethnic minorities comprise 21% of the US population over age 65 [26]. This diversity suggests interventions effective for one portion of the aging population (eg, Hispanic adults over 80) may not generalize to other segments (eg, African-American adults under 65). Tailoring interventions offers one approach for ensuring instructional content matches each participant’s specific characteristics. Tailoring is “any combination of strategies and information intended to reach one specific person, based on characteristics that are unique to that person, related to the outcome of interest, and derived from an individual assessment” [27]. Tailored interventions have outperformed non-tailored interventions for participants with type 2 diabetes, hypertension, and physical activity [28,29]. However, no known study investigated tailored eHealth literacy interventions, indicating a significant opportunity exists for improving the efficacy of interventions.

A systematic review of eHealth literacy interventions for older adults can provide a foundation for improving intervention outcomes. A recent systematic review investigated health literacy interventions for older adults [11] but excluded large-scale experimental eHealth studies for older computer learners (eg, Xie [18-20]) and sampled only computer literate older adults [11]. Similarly, a prior review examined online health literacy interventions but is distinct from this systematic review in several key aspects [14]. First, the Car et al [14] review included only studies using randomized controlled trials (RCTs) or controlled before and after studies (CBA). In comparison, our systematic review analyzed a more comprehensive sample of studies including but not limited to RCTs or CBAs. Our broad scope is justified given the small number of existing studies on this topic. Second, the Car et al [14] review included only two studies in their study sample. This small sample size led Car et al [14] to conclude that they could not “draw any conclusions about the implications of [their systematic review] for the content or delivery of consumer Internet skill interventions” [14]. Third, the Car et al [14] review applied no exclusion criteria for study participants. In contrast, this systematic review focuses explicitly on the older population and excludes studies with participants age 50 and below. Our systematic review thus makes new contributions to the literature.

We address a significant gap in the literature by providing a more comprehensive review that includes eHealth literacy interventions with both computer literate and illiterate older adults. This approach is necessary because (1) many older adults lack computer literacy [7], (2) excluding computer illiterate older adults may exclude studies with SES participants because of this population’s low computer literacy levels [5], and (3) limiting the review to eHealth literacy interventions excludes health literacy interventions that use ICTs but do not use the term eHealth (eg, Neafsey et al [30]). The following research questions guide this review: (1) What intervention strategies have been used to improve older adults’ eHealth literacy?, (2) What strategies are found to be effective in improving older adults’ eHealth literacy?, and (3) What evidence supports the effectiveness of eHealth literacy interventions for older adults?

## Methods

### Article Selection

We performed four rounds of systematic selection in January 2013 to identify relevant articles: (1) database selection, (2) keyword search, (3) screening the titles and abstracts, and (4) screening the full text.

#### Round 1: Database Selection

We conducted search queries with electronic databases accessible at the University of Texas at Austin. Database selection involved two steps. First, we identified academic fields pertinent to the literature review, resulting in a list of nine fields with a total of 159 databases (see Multimedia Appendix 1 for the identified fields). Second, we evaluated the databases for these fields with inclusion criteria to confirm their relevance (see Multimedia Appendix 2 for inclusion criteria). This process produced a set of 28 databases (see Multimedia Appendix 1 for selected databases).

#### Round 2: Keyword Search

The following keywords were used to search the 28 selected databases: “health literacy” OR “eHealth literacy” OR “e-Health literacy” OR “information literacy” OR “computer literacy” AND “old* adult*” OR “senior*” OR “elder*” OR “aging” OR “ageing” OR “babyboomer*” OR “retiree*”.

To ensure an inclusive selection of results, we applied no additional limiting criteria in the second round. Due to differences among the 28 databases, the keywords were used to search the articles’ full text, abstract, or title. This process produced a total of 253 articles.

#### Round 3: Screening the Titles and Abstracts

One author (IW) screened the titles and abstracts for the 253 articles to ensure each study included *older adults* and involved an *eHealth literacy* intervention*.* Round 3 produced 30 articles that met the following criteria:

1. *Older adults* must make up a significant proportion of study participants. For the purpose of this study, older adults are defined as individuals age 50 years and above. This definition expands the scope of the review and is consistent with growing appreciation of the role that health behavior interventions play in healthy aging for those under age 65 [31]. Studies including no older adults in their samples (eg, Cormier et al [32]) were eliminated.

2. The study involved evaluation of an *intervention* using empirical data. To expand the scope of the review, we included qualitative studies and studies with a non-experimental research design, provided that these studies evaluated an intervention. Studies not reporting original and empirical data, such as literature reviews [11] (eg, Echt [33]), were excluded from our sample.

3. The intervention must have focused on improving *eHealth literacy* or improving a health outcome by improving *eHealth literacy*. To be more inclusive, we included interventions focused on health literacy related to specific health conditions, such as mental health literacy (eg, Walker [34]) or oral health literacy (eg, Hjertstedt, Barnes, Sjostedt [35]), or a single condition, such as ulcers (eg, Hartigan, Murphy, Hickey [36]).

#### Round 4: Screening the Full-Text

**Figure 1:**
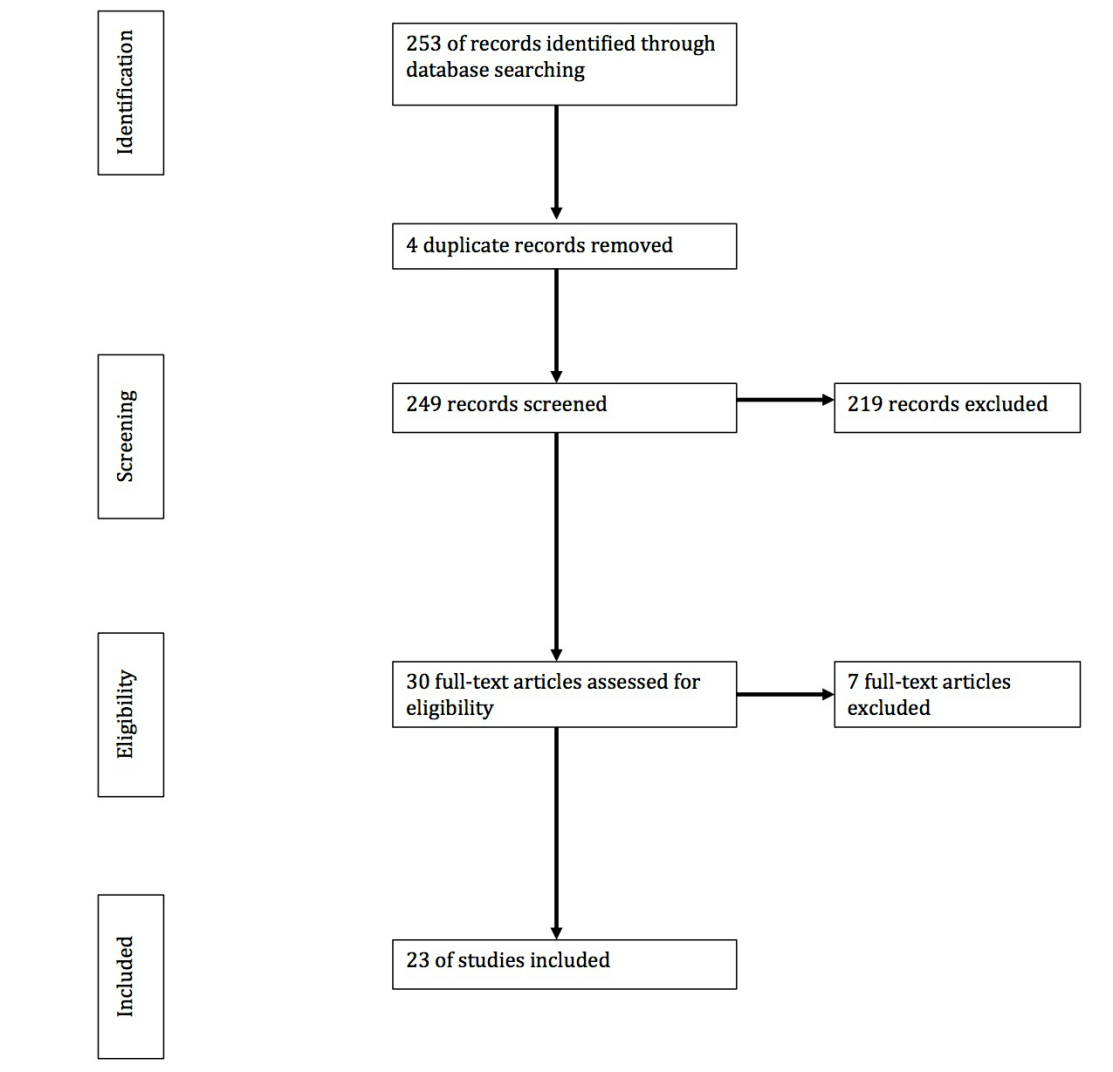
Four-round selection process.

One author (IW) reviewed the full text of the remaining 30 articles to confirm consistency with the three criteria applied to review article titles and abstracts during the third round. We eliminated another 7 articles in Round 4. Articles were eliminated for not reporting empirical data [37-39], not including older adults [40,41], not focusing on health literacy [42], or providing no information on the intervention content or materials [43]. The final sample contained 23 articles. Figure 1 summarizes this four-round selection process.

## Results

### Overview

The 23 articles were published between 2003 and 2013 and report results from 23 independent studies [18-20,30,34-36,44-59]. These articles’ key characteristics appear in Multimedia Appendix 3. We report below these articles’ use of theory (or a lack of it), research design, measures, participant characteristics, outcomes, intervention materials, and whether or not they involved tailored interventions.

### Themes That Emerged From Key Findings

#### Intervention Strategies for Improving Older Adults’ eHealth Literacy

We identified several intervention strategies among the sampled studies, including collaborative learning and tailored intervention content. Collaborative learning “involves the construction of meaning through interaction with others and can be characterized by a joint commitment to a shared goal” [60]. Three studies [18-20] used collaborative learning strategies to teach participants eHealth literacy.

Similarly, four studies (17%) tailored intervention content [30,44,45,61]. Tailored intervention materials “are intended to reach a specific person, are based on characteristics that are unique to that person, are related to the outcome of interest, and have been dervied from an individual assessment” [27]. Of the four studies, three studies disseminated tailored content to participants in print [30,44,45], while one study disseminated tailored content by telephone [61].

#### Effectiveness of Strategies

Interventions applying collaborative learning strategies significantly improved participants’ computer and Web knowledge and skill [18,20], eHealth literacy self-efficacy [18,19], and eHealth literacy skill [19]. Similarly, findings from the four studies that applied tailoring as an intervention strategy provided evidence for the effectiveness of this strategy [30,44,45,61]. These studies found that tailored educational interventions significantly improved participants’ blood pressure control [30,61], medication self-efficacy [30], and medication adherence [44,45].

#### Evidence Supporting the Effectiveness of eHealth Literacy Interventions for Older Adults

The sampled studies provided evidence for the effectiveness of eHealth literacy interventions for older adults. The studies that conducted RCTs all found significant improvement for outcome measures from pre- to post-intervention [18,20,34,44,46,47,61]. Likewise, the studies using a one group, pretest and posttest research design all identified significant improvements for outcome measures [19,30,35,36,48-54]. The remaining five studies [45,55-58] used a variety of research designs (eg, quasi-experimental design [56] and post-hoc analysis of an RCT [45]) or relied on qualitative data [55]. Each of these studies found evidence supporting the effectiveness of eHealth literacy interventions for older adults [45,55-58]. See Multimedia Appendix 3 for key findings from each of these studies.

### Use of Theory

About half of the studies (12/23, 52%) applied no theoretical framework. The 11 studies that applied a theoretical framework drew on two fields: (1) health behavior (seven studies) and (2) education (4 studies). Of these 11 studies, 7 (30%) used health behavior theories: three studies (13%) used the Transtheoretical Model [34,50,55], two studies (9%) used the Health Belief Model [46,61], and two studies (8.7%) used Social Cognitive Theory [30,49]. Of the four studies (17%) using a learning theory, three studies (13%) used Social Interdependence Theory [18-20], one study (4%) used a Cognitive Theory of Multimedia Learning [20], and one study (4%) used Transformative Learning [51].

### Research Design

The studies used a variety of research designs, consistent with the broad selection criteria used in our searches. Just under half (11/23, 48%) used designs with pretest and posttest of a single condition [19,30,35,36,48-54], while one study used a quasi-experimental design [56]. RCTs defined as “trials that include at least one experimental condition, along with a control condition, and randomize the assignment of participants to a condition” [59], were used in seven studies (30.4%) [18,20,34,44,46,47,61]. One study (4.3%) conducted a post-hoc analysis of RCT data [45], while another study (4.3%) was an observational study that used survey questionnaires and in-depth interviews to evaluate participants from the experimental group of an RCT [55]. The two remaining studies (9%) were cross-sectional survey studies [57,58].

### Health Literacy Measures

Five studies (22%) used either the Test of Functional Health Literacy in Adults (TOFHLA) or the Short Test of Functional Literacy in Adults (S-TOFHLA) [45-47,50,51], two studies (9%) used a version of the Rapid Estimation of Adult Literacy in Medicine (REALM) [35,61], and three studies (13%) used the eHealth Literacy Scale (eHEALS) [18-20]. More than half (13/23, 57%) used no standardized or validated instrument to measure literacy [30,34,36,46,48,49,52-58].

### Sample Characteristics

Sample size varied between 11 participants in a pilot study and 909 participants for an RCT (a notable outlier is Olson, Sabogal, and Perez [56], which examined secondary survey data collected from 57,104 Medicare beneficiaries). More than half of the studies (13/23, 57%) had over 100 participants [18-20,34,45-47,49,52-56,61], five studies (22%) had between 30 and 99 participants [35,36,48,57,58], and four studies (17%) had fewer than 30 participants [30,44,46,51]. One study reported the percentage of participants for two age ranges, but did not report the total sample size [54]. About one third (34%) of that study’s participants were 65-79 years, while another 41% were over age 80 [54]. Overall, participant age varied considerably across the studies, with mean participant age ranging between mean 61 and mean 84. Many studies (17/23, 74%) were majority female [18-20,30,34-36,48-58]. Of studies reporting race or ethnicity, five studies (22%) reported majority African-American participants [18-20,49,51], four studies (17%) reported majority white participants [30,35,50,56], and one study reported a Latino/Hispanic majority [52]. The six studies (26%) with majority racial/ethnic minority participants targeted older adults of lower SES or a specific racial/ethnic minority group in the United States (one exception is Williams, Manias, Liew, Gock, Gorelik [46], who focused on Greek and Italian immigrants to Australia).

Only seven studies (30%) reported data on participants’ income [18-20,45,49,50,61]. Of those studies, four studies reported that at least 20% of their participants earned less than $20,000 per year [18-20,49], one study reported that 71% of participants earned less than $25,000 per year [50], one study reported that 37% of participants had “low household income” [45], and one study reported that 19% of participants had “inadequate incomes” [61]. Only 11 studies (48%) reported data on educational attainment [18-20,30,34,35,45,47,50,51]. Of those studies, six reported that more than 10% of participants had less than a high school education [18,30,35,45,50,51], three studies reported less than 10% of participants had less than a high school education [19,20,49], two studies reported a mean of more than 10 years of formal education [34,47], and one study reported that 23.9% of participants had a high school education or below [35].

### Outcomes of Interest

Ten studies (43%) targeted a specific health outcome or behavior as the outcome of interest, with health literacy serving as an independent variable [30,45-47,53,55,56,61]; 13 studies (57%) targeted some form of literacy as an outcome of interest. Of these studies, eight studies (35%) targeted eHealth literacy [18-20,48,49,54,57,58] while five studies (22%) targeted health literacy [34-36,51,52]. Of the 10 studies targeting a health outcome or behavior with health literacy serving as an independent variable, four studies (17%) targeted medication management [45-47], two studies (9%) targeted hypertension management [30,61], two studies (9%) targeted diabetes management [55,56], and one study (4%) each targeted mental health [53] and pharmacist-patient communication [50].

### Intervention Materials

Most (6/8) of the eHealth literacy interventions used instructional materials developed by the National Institute on Aging (NIA) of the National Institutes of Health (NIH) [18,19,48,49,57,58]. Additionally, one study used a multimedia tutorial developed by the National Library of Medicine of the NIH [20], and one study developed a website with stroke information through a collaborative partnership between various Pennsylvania healthcare providers [54]. The six studies that used NIA materials all adapted materials from the NIH Senior Health “Training the Trainers Toolkit”, freely available on the NIH Senior Health website [18,19,48,49,57,58]. This toolkit provides lesson plans for instructing older adults on how to locate reliable health information online using desktop or laptop computers [62]. In contrast with the uniformity among the eHealth literacy studies in terms of the instructional materials they used in their interventions, the 15 health literacy interventions drew on a wide range of materials, such as those developed by non-profits or researchers themselves.

### Research Setting and Location

Fourteen studies (61%) occurred in informal learning settings (eg, public libraries or senior centers) [18-20,35,47-54,57,58]; four studies (17%) in clinical settings [34,44,45,61], four studies (17%) were administered remotely via ICTs including three by telephone [34,55,61] and one by tablet computer [30]). One study (4%) involved an intervention carried out via broadcast public service announcements on radio and television [56]. Data collection for 18 studies (78%) took place in the United States [18-20,30,35,48-52,54,56,59,61-65], three studies (13%) in Australia [34,46,53], one study (4%) in England [55], and one study (4%) in Ireland [36].

## Discussion

### Principal Findings

eHealth literacy interventions can provide older adults with the skills and knowledge necessary to benefit from eHealth resources [18-20]. However, this review highlights the need for theory-based interventions that apply high-quality research design. The eHealth interventions in our final sample most closely aligned with the public health perspective of health literacy [16], a trend not identified by prior reviews. These interventions were consistent with the public health perspective in that they viewed eHealth literacy as an asset that increases individuals’ ability to access, assess, understand, and apply health information to make health-related decisions [16]. These eHealth literacy interventions targeted eHealth literacy as an outcome of interest, similar to how health literacy interventions consistent with the public health perspective target health literacy as an outcome of interest [16]. This approach could potentially address a vital need among underserved segments of the older population, such as those with low SES, that are most likely to have poor health literacy [5]. However, of the eight studies with eHealth literacy as an outcome of interest, only those conducted by Xie [18-20,49] reported data on either participants’ income or education, making it difficult to determine whether the other eHealth interventions targeted low SES participants.

The sampled eHealth interventions were inconsistent with the clinical perspective in that none of the interventions included a health outcome as an outcome measure [16]. Including health outcomes as outcome measures is important because this can clarify the relationship between eHealth literacy and health outcomes for older adults. Another characteristic of the clinical perspective is that interventions are evaluated in clinical settings [16]. None of the sampled eHealth interventions occurred in a clinical setting. Investigating eHealth interventions in clinical settings could generate important knowledge by removing environmental distractions, such as noise, that can occur in informal learning settings such as a public library [18-20]. Additionally, clinical settings present distinct challenges, such as reliably measuring health literacy without causing patients embarrassment, stress, or discomfort [63]. Objective measures of eHealth literacy skill, such as those used by Xie [20], could be difficult to administer in clinical settings because they require participants to use a computer. Subjective measures, such as the self-reported eHealth literacy scale (eHEALS) [64], offer an alternative but must be updated for new Internet technologies, such as social media, to ensure their validity [17,65].

Similarly, developing and applying theory could enhance the quality of research on eHealth literacy interventions. Theory development advances emerging fields by shifting the research focus away from simply discovering new facts to explaining facts, predicting outcomes [66], and generalizing results [67]. A review examining the use of theory in the emerging field of Web 2.0 found the limited use of theory slowed the advancement of scientific knowledge on Web 2.0-associated social phenomena [68]. Consistent with prior reviews of health and eHealth literacy [13], most studies in this review applied no theory. Among the theory-based studies, eHealth literacy studies used only learning theory (eg, Xie [18-20]), while health literacy interventions used various health behavior theories (eg, Miller et al [50], Bosworth et al [61]). The limited range of theories for eHealth studies likely resulted from the small number of researchers investigating eHealth literacy interventions for older adults. As the number of researchers contributing to the field grows, greater variability in the application of theory may be expected.

None of the sampled studies used an eHealth literacy-specific theoretical framework, such as the Lily model or Chan and Kaufman’s [6] proposed framework. As a relatively new construct, it is not surprising that only a limited number of theoretical frameworks for eHealth literacy has been proposed. However, hypothesizing and testing the relationship between theoretical constructs is essential to theory development in intervention research [69]. As a result, using eHealth literacy-specific theoretical frameworks to guide eHealth intervention research with older adults can support the development and improvement of interventions. Theoretical development will be especially important as mobile technologies, such as smartphones and tablet computers, continue to grow as a source of digital health information [1].

While the Lily model describes the skills and knowledge necessary for eHealth literacy, it includes no cognitive, social, or environmental variables and lacks empirical validation. The Chan and Kaufman [6] framework mapped cognitive demands onto this model but also lacks empirical validation. Combined, these two models demonstrate a need for future eHealth literacy research to empirically evaluate eHealth literacy theories. Such an evaluation is necessary to determine the extent to which these models generalize to the older population.

Along with a lack of theory, poor research design makes evaluating intervention outcomes problematic. Consistent with recent health literacy reviews [11,13], most sampled studies used non-experimental, cross-sectional, or quasi-experimental designs that tested a single condition without a control condition. RCTs, known to produce the highest quality evidence in health-related research by systematically limiting potential biases [70], are used in only a few studies. Several studies also used post-intervention surveys to assess outcomes (eg, Susic [57]). These surveys used only self-reported data without objective measures of eHealth literacy. Recent reviews found the lack of standardized health literacy measures decreases the generalizability of findings from health literacy interventions [11,13]. A similar issue emerged in the results from this review, where over half of the examined studies lacked measures of eHealth literacy. Further, several studies measured a health outcome without measuring eHealth literacy. For example, a mental health literacy intervention with community-dwelling older adults measured depression and physical activity (the outcomes of interest) but did not measure eHealth literacy as an outcome [34]. As a result, the study offers little information on the relationship between eHealth literacy and health outcomes.

A notable distinction between health literacy and eHealth literacy appeared in the intervention materials. The health literacy interventions used various materials, including condition-specific materials, such as an ulcer pamphlet [36]. In contrast, eHealth literacy interventions used NIH materials, with the exception of Gross et al [54], which used materials developed locally. Of studies using NIH materials, only Xie [20] did not use the NIH Senior Health training materials (that study used a tutorial developed by the National Library of Medicine of NIH). Again, this uniformity reflects eHealth literacy’s status as an emerging field studied by a handful of researchers—Xie [18-20,49] alone conducted half of the sampled eHealth literacy studies. This uniformity raises several issues. First, the NIH Senior Health materials teach skills and knowledge specific to desktop or laptop computers. The contemporary ICT environment features a growing variety of devices, including smartphones and tablets, and almost one third of adults access health information on a mobile device [1]. As a result, literacy with these newer devices will become increasingly important. Second, the NIH Senior Health materials do not address Web 2.0 or social networking applications. Adults increasingly go online to connect with others over health issues, such as soliciting peers for health advice [1]. Ignoring the impact of new ICTs on health—and the associated skills required to use them—could negatively impact older adults’ eHealth literacy. Last, the NIH Senior Health materials teach eHealth as a general skill applicable to many health issues. Teaching eHealth literacy for specific health issues of particular interest to individuals could potentially improve learning by making intervention content more personally relevant. Only one eHealth literacy intervention focused on a specific health issue—stroke [54]. That intervention, however, did not report pretest and posttest data for participants’ stroke knowledge, so the intervention’s effectiveness is unclear.

The studies examined in this systematic review included participants that varied considerably in age, but most of these studies did not report participants’ income, education, race, or ethnicity. Mean participant age ranged from 61-84 years, raising the question of whether interventions effective for the “younger” segment of the older population can generalize to the “oldest” old. The failure to report participants’ income or education is problematic given the low median income of older adults in the United States [26,71] and the association of low SES with poor health literacy [9]. As with age, interventions effective for one group may not generalize to others, so reporting participants’ income, education, race, or ethnicity is essential to understanding the implications of an intervention’s results.

Tailoring offers a solution for addressing the influence of individual characteristics on health outcomes. While tailoring has been proven effective in health interventions [29,72], only four of the sampled studies involved tailoring. For eHealth literacy interventions with older adults, tailoring could adjust intervention content for factors like participants’ computer experience, health literacy, income, educational attainment, age, race, ethnicity, language, or health issues. However, tailoring requires knowledge of how these factors affect outcomes. As noted above, most studies did not report data on participants’ income, education, race, or ethnicity. Also, administering individual assessments necessary to tailoring typically demands significant resources [28]. New ICTs, such as tablet computers, can provide just-in-time intervention content based on individuals’ reported behavior, though applications for new ICTs, such as tablets, can also present potential usability challenges for older adults [73]. Among the sampled studies, only one used tablets [30]. Notably, that study collected participant data using tablets but disseminated the tailored intervention content in print.

### Limitations and Future Directions

This systematic literature review contains several limitations. The keyword search did not use a controlled vocabulary (eg, Medical Subject Headings, Cumulative Index to Nursing and Allied Health) and was restricted to the title, keywords, and abstract for each article. This inclusion criterion may have excluded studies that deal with aspects of eHealth literacy but do not contain these exact keywords we used. Only studies with full text written in English were included in the sample, which excluded articles in non-English journals. Also, this review did not include studies that may have contributed to the development of the eHealth literacy construct (that pre-dated it and thus did not use the exact terms to be included in our searches). Nonetheless, this review identified important gaps in the literature that require future research. These gaps include (1) What is the relationship between eHealth literacy and health outcomes for older adults?, (2) Which theoretical frameworks are effective for developing and assessing eHealth literacy interventions for older adults, and which are more effective than others in what context?, (3) What is the relationship between eHealth literacy interventions conducted in clinical settings and those conducted in informal learning settings, and what factors should be considered when implementing eHealth literacy interventions in clinical settings?, (4) What instructional materials best facilitate older adults’ improvements in their eHealth literacy, and how can materials stay current given ICTs’ rapid development?, and (5) Which individual characteristics, such as health literacy level, computer experience, or SES, should be considered in interventions to tailor the health content and delivery strategies?

### Conclusions

This paper reports findings from a systematic review of 23 articles on health literacy interventions and eHealth literacy interventions for older adults drawn from 28 relevant databases in nine fields. The eHealth literacy interventions in the sampled articles used eHealth literacy as an outcome of interest, applied learning theories, and occurred in informal learning settings such as senior centers and public libraries. In contrast, health literacy interventions (that involved ICTs as a key aspect of their interventions) often targeted specific health outcomes, applied health behavior theories, and occurred in both informal learning and clinical settings. These results indicate a significant gap in the literature on eHealth literacy interventions that use health outcomes for outcome measures. Additionally, most of the studies used no theoretical framework, and only seven studies were RCTs. These results highlight a great need to develop and assess theory-based interventions applying high-quality research design.

## Multimedia Appendix 1

Selected databases organized by field.

## Multimedia Appendix 2

Database inclusion criteria.

## Multimedia Appendix 3

Selected articles.

## Abbreviations

CBA: controlled before and after studies
CTML: cognitive theory of multimedia learning
ICT: information and communication technology
NIH: National Institutes of Health
RCT: randomized controlled trial
SES: socioeconomic status
SIT: social interdependence theory
