# Supplementary material for: eHealth Literacy Interventions for Older Adults: A Systematic Review of the Literature
Source: J Med Internet Res. 2014 Nov 10;16(11):e225. doi: 10.2196/jmir.3318 (PMC4260003; doi:10.2196/jmir.3318)
Supplement: Supplementary file 1 [file jmir_v16i11e225_app1.pdf]

Multimedia Appendix 1. Selected databases organized by field.

Field		Database
Anthropology	1.	Anthropology Plus
	2.	AnthroSource
	3.	JSTOR
Communication	4.	Communication & Mass Media Complete (CMMC)
Computer	5.	ACM (Association for Computing Machinery) Digital
Science	6.	Library
	7.	CiteSeer^x
	8.	Computer Source
	9.	IEEE Xplore
	10	INSPEC
	.	Internet and Personal Computing Abstracts
	11	Science & Technology Collection
	.	
Consumer Health	12	Alt HealthWatch
	.	CINAHL (Cumulative Index to Nursing & Allied
	13	Health) Plus with Full Text
	.	Consumer Health Complete
		Health Reference Center
	14	Health Source: Consumer Edition
	.	
	15	
	.	
	16	
	.	
Education	17.	Education Full Text
	18	Education Resources Information Center (ERIC)
	.	
Library and	19	Emerald Insight
Information	.	Information Science & Technology Abstracts (ISTA)
Science	20	Library, Information Science & Technology Abstracts
	.	(LISTA)
	21	Library Literature & Information Science Full Text
	.	

	22.	
Medicine	23.	Health Source: Nursing/Academic Edition
	24.	PubMed
Psychology	25.	PsycARTICLES
	26.	Psychology & Behavioral Sciences Collection
	27.	PyscINFO
Sociology	28.	Wiley Online Library
