# Supplementary material for: eHealth Literacy Interventions for Older Adults: A Systematic Review of the Literature
Source: J Med Internet Res. 2014 Nov 10;16(11):e225. doi: 10.2196/jmir.3318 (PMC4260003; doi:10.2196/jmir.3318)
Supplement: Supplementary file 2 [file jmir_v16i11e225_app2.pdf]

Multimedia Appendix 2: Database inclusion criteria

The inclusion criteria for databases required that databases include journals publishing peer-reviewed research articles; this criterion excluded databases limited to graduate dissertations or theses, non-peer-reviewed periodicals, news, or non-print media (e.g., video or audio).

1) Permit searches of full text, abstract, or keywords.
2) Full text published in English.
3) Exhibit relevance to the subject (e.g., a database specific to programming languages in the computer science field was excluded).

Subsequent rounds used these twenty-eight databases to locate relevant research articles.
