# Supplementary material for: eHealth Literacy Interventions for Older Adults: A Systematic Review of the Literature
Source: J Med Internet Res. 2014 Nov 10;16(11):e225. doi: 10.2196/jmir.3318 (PMC4260003; doi:10.2196/jmir.3318)
Supplement: Supplementary file 3 [file jmir_v16i11e225_app3.pdf]

Multimedia Appendix 3. Selected articles.

Author, Year	Publication venue	Intervention	Research topic	Sample size/age	Participant location	Research design	Key finding
Aspinall (2012) [49]	Med Ref Serv Q	Two workshops: 1. Communicating with health care providers 2. Online health information search (adapted from NIHSeniorhealth/ Medlineplus, based on needs assessment/focus groups)	eHealth literacy among older adults.	n=52; 55-100	United States	Pre- and post-test survey assessments (pilot study with a single condition).	Identified issues include: 1) covering drug information; 2) using a Likert scale pre- and post-test; and 3) making the post-test questionnaire a survey to increase response.
Bosworth (2009) [44]	Ann Intern Med	Three intervention conditions: 1. Tailored blood pressure self-management intervention delivered bi-monthly over telephone by a nurse administrator. 2. Home BP monitoring. 3. 1 and 2 combined.	Hypertension self-management behavior.	n=636; mean 61	United States	RCT (2 X 2) with four conditions (three intervention conditions and a control).	Combination of the tailored educational intervention and the home BP monitoring significantly improved patient blood pressure control compared to the control condition.

Gattuso (2003) [54]	Australas J Ageing	8 sessions focused on 1) information about depression; 2) skills development; and 3) social support.	Mental health literacy for older adults with age-related stress.	n=103	Australia	Pre- and post-intervention design with a 6-month follow-up (pilot study with a single condition).	Significant reduction in depression pre- to post-intervention, maintained after 6 months.
Gross et al (2007) [55]	J Consum Health Internet	Two intervention conditions:<br>1. Online;<br>2. In-person;<br>Interventions used same content on:<br>1. Stroke information;<br>2. Locating stroke information online;	Stroke-specific eHealth literacy.	n=not reported; 65-79	United States	Pre- and post-intervention design with two conditions (conditions were analyzed together as a single condition).	Increases in: 1) knowledge of Internet resources; 2) perceived ability to find stroke information online;
Hartigan et al (2011) [36]	Int J Older People Nurs	Participants given a leaflet on pressure ulcer prevention for one week.	Pressure ulcer prevention knowledge.	n=75; 66-99 (mean 79.9; SD 6.5)	Ireland	Pre- and post-intervention design with a single condition.	Pressure ulcer prevention knowledge increased pre- to post-intervention
Hjertstedt et al (2012) [35]	Gerodontology	Five 2-hour discussions between a participant and two dental students on	Oral health literacy and oral hygiene of older adults.	n=67; mean 84; SD 7.3	United States	Pre- and post-intervention design with a	Significant increase in oral health literacy pre- to post-

		oral health literacy and hygiene.				single condition.	intervention.
Long et al (2011) [56]	Health Expec	Structured questioning over the phone by nurses to motivate diabetes management, guided by tailoring software. Call frequency dependent on glucose control.	Promoting health literacy for type 2 diabetics using tele-care.	n=156; Median age=67 (no reported mean)	England	Pre- and post-survey of participants in the intervention condition of a RCT; in-depth interviews.	Potentially effective for promoting self-efficacy for glucose control and increasing health literacy.
Miller et al (2008) [51]	J Am Pharm Assoc	Presentation of “Ask Me 3” educational program adapted to patient-pharmacist communication. Program teaches participants three specific questions to ask their pharmacist.	Health communication between community-dwelling older adults and pharmacists.	n=106; mean 75.1; SD 7.5	United States	Pre- and post-intervention design with a single condition.	Significant increase in participants planning to bring current list of medications to pharmacist visits.
Morrow et al (2007) [48]	J Aging Health	Two conditions for pharmacy instructions: 1) patient-centered (eg, larger font; improved readability); 2) standard (control).	Patient-centered pharmacy instructions for patients with Chronic Heart Failure.	n=236	United States	RCT with intervention and control conditions (this study reports on one part of an intervention)	Patient-centered instruction preferred for learning about medication adherence; standard instruction preferred drug interaction

							knowledge.
Neafsey et al (2008) [30]	Comput Inform Nurs	Computer program identifies high priority medication interactions for a patient, then provides tailored educational content on the interactions using the tablet.	Tablet-computer based, tailored health literacy tutorial for hypertensive older adults.	n=11; 65-98 (mean 80.36; SD 8.52)	United States	Beta-testing of a computer program (measures taken once per month for four months from a single condition).	Increase in knowledge, medication self-efficacy, and reduction in blood pressure from the first to last measure. High satisfaction with the program.
Noureldin, et al (2012) [46]	Pharmacotherapy	Three part tailored intervention: 1) patient education on their prescription; 2) therapeutic monitoring with a pharmacist; 3) physician communication.	Medication adherence for older adults that have low health literacy.	n=281 (mean 63; SD 9)	United States	Post-hoc analysis of RCT with intervention/ control conditions.	Pharmacists may affect how health literacy influences drug adherence for patients with heart failure.
Ntiri et al (2009) [52]	Gertontol Geriatr Educ	"Transformative Learning Intervention" emphasizing interaction communication about diabetes knowledge over six sessions.	Functional health literacy/diabetes knowledge among African-American older adults.	n=20; 57-84 (mean 68.1)	United States	Pre- and post-intervention design with a single condition.	Diabetes knowledge, health literacy increased significantly pre- to post-intervention. No increase in diabetes literacy.
Olson et	Am J Public	Holistic intervention	Diabetes	n=57,104	United States	Quasi-	Disparities in

al (2008) [57]	Health	with two parts: 1) media campaign on Spanish radio stations; 2) diabetes self-management materials	management for older Latinos on Medicare.			experimental design comparing 1) Hispanic and 2) White Medicare beneficiaries.	hemoglobin testing decreased between Hispanics and Whites.
Ownby et al (2012) [45]	Clin Gerontol	Two interventions: 1) automated reminding: automated daily phone calls to take medication. 2) tailored information on memory problems and aging provided	Medication adherence among older adults (including those with memory problems).	n=27; 71-92 (mean 79.93)	United States	Pre- and post-intervention design (random assignment to two intervention and one control conditions).	Automated reminding and tailored information effective for increasing medication adherence.
Strong et al (2012) [59]	New Libr World	Taught seven modules from “Helping Older Adults Search for Health Information Online” tutorial developed by NIH. Developed personal health information records with librarians.	eHealth literacy intervention for older adults.	n=50	United States	Survey taken pre- post- and 6 week post-intervention.	Participants reported increased use of health databases post-intervention, 70% of participants created electronic health records.
Susic, J.	J Consum	Librarian taught	eHealth literacy	n=60;	United States	Non-	Increased

(2009) [58]	Health Internet	"Introduction to NIHSeniorHealth" module developed by NIH.	intervention for older adults.	mean 70		experimental (no pre-intervention testing; post- and 6-month follow up testing)	participants' use of NIHSeniorhealth to locate health information.
Valle et al (2006) [53]	Clin Gerontol	Two sessions and two print, Spanish language, fotonovelas focused on Alzheimer's: 1) service access/use 2) the diagnosis process	Alzheimer's educational intervention for older adults with low knowledge of Alzheimer's.	n=111; mean 68.8; SD 8	United States	Pre- and post-intervention design with a single group	Intervention effective for participants with low-knowledge about Alzheimer's disease.
Walker et al (2010) [34]	Br J Psychiatry	Ten mental health literacy modules delivered by mail over twenty-four months. Telephone calls used to motivate participants to use the modules.	Mental health literacy intervention to prevent depression among older adults.	n=909; 60-74	Australia	RCT with 2 x 2 x 2 factorial design: (folic acid + B12 vs placebo) x (physical activity vs nutrition promotion control) x (mental health literacy vs pain control information).	Intervention ineffective for reducing depression.

Williams et al (2012) [47]	Ren Soc Australas J	Intervention included: 1) individualized medication review with a nurse; 2) PowerPoint presentation on medication management; 3) booklet based on presentation 4) motivational phone calls (bi-monthly).	Medication self-management among culturally and linguistically diverse people with diabetes, cardiovascular disease, and kidney disease.	n=29; mean 74.31; SD 8.37	Australia	Pilot study for RCT with two conditions: 1) multifactorial intervention 2) control condition.	Intervention infeasible due to high attrition. Additional interpreters and additional consultation time necessary.
Xie, B. (2012) [50]	Libr Inf Sci Res	Eight modules from "Helping Older Adults Search for Health Information Online" tutorial developed by NIH.	eHealth literacy intervention for older adults using individualistic learning.	n=218; 60-89 (mean 70.0; SD 8.7)	United States	Pre- and post-intervention design with a single condition.	Intervention effective for increasing computer and Internet knowledge, attitudes towards computers.
Xie, B. (2011a) [18]	J Med Internet Res	Four modules from "Helping Older Adults Search for Health Information Online" tutorial developed by NIH to increase older adults' eHealth literacy were	Evaluating the effectiveness of collaborative vs individualistic learning for teaching older adults eHealth literacy.	n=146; 56-91 (mean 69.99; SD 8.12)	United States	Randomized, 2 X 2 mixed factorial design: Between-participants variable: collaborative	Intervention effective for increasing computer and Web knowledge/skill, along with eHealth literacy

		taught using either collaborative or individualistic learning.				vs individualistic; Within-participants variable: time of measurement.	efficacy. No difference for learning method.
Xie, B. (2011b) [20]	J Am Soc Inf Sci Technol	NIH tutorial on evaluating health information presented in four conditions: 1) individualistic/visual 2) individualistic/visual + audio narration 3) collaborative/visual 4) collaborative/visual + audio narration	Evaluating effect of collaborative vs individualistic learning and presentation channel (visual vs visual + auditory) for teaching older adults eHealth literacy.	n=124; mean 68.15; SD 9	United States	Randomized, 2 x 2 x 2 mixed factorial design: Between-subjects variables: 1) learning method 2) presentation channel Within-subjects variable: time of measurement	No significant effect for learning method or presentation channel, but significant increases in eHealth literacy skills and efficacy.

Xie, B. (2011c) [19]	J Am Soc Inf Sci Technol	Adapted and tested eight modules from “Helping Older Adults Search for Health Information Online: A Toolkit for Trainers” developed by NIH.	eHealth literacy intervention for older adults using collaborative learning.	n=172; 52-91 (mean 70.4; SD 8.0)	United States	Pre- and post-intervention design with a single condition.	Collaborative learning effective for increasing computer and Internet knowledge, along with attitudes towards eHealth literacy.
